# Enhancing Cervical Foraminotomy Outcomes With Patient-Specific 3D Anatomical Models: A Comparative Study

**DOI:** 10.7759/cureus.76426

**Published:** 2024-12-26

**Authors:** Michelle Ho, Ali Ravanpay, Sarah Bastawrous, Kevin Chorath, Lei Wu

**Affiliations:** 1 Diagnostic Radiology, University of Washington, Seattle, USA; 2 Neurological Surgery, University of Washington, Seattle, USA

**Keywords:** 3d model, education tool, fluoroscopy time, minimally invasive posterior cervical foraminotomy, pre-surgical planning, surgical tool

## Abstract

Introduction: Cervical foraminotomy is a procedure used to treat patients with radiculopathy. While the procedure can be performed using a minimally invasive technique, achieving complete visualization of relevant anatomy can be challenging. This study explores the use of patient-specific three-dimensional (3D) printed anatomical models, created from advanced medical imaging data, for preoperative planning and intraoperative guidance in cervical foraminotomy by comparing fluoroscopy time, operative time, estimated blood loss volume, and functional improvement.

Methods: We conducted a retrospective case-controlled study comparing patients who underwent cervical foraminotomy with the aid of a 3D model to those who received standard of care.

Results: Our findings indicate a statistically significant reduction in fluoroscopy time for the 3D model group (21.8 seconds vs 49.0 seconds, p<0.05). Although functional outcomes for the 3D model group showed a trend towards significance (p=0.14), no significant differences were observed in operative time or estimated blood loss (p=0.25 and p=0.31, respectively).

Conclusion: These results suggest that patient-specific 3D models can enhance the understanding of complex anatomical structures and may improve surgical outcomes in cervical foraminotomy.

## Introduction

Three-dimensional (3D) printed anatomical models created from advanced medical imaging data are increasingly being used in healthcare for preoperative planning [1-3]. 3D printed models allow surgeons to plan surgical approaches, identify potential hurdles, and rehearse cases [4,5]. Having patient-specific 3D models can also help surgeons appreciate complex 3D relationships between anatomic structures, particularly when operating within a limited surgical window [6,7]. In addition, for tumor resections where the tumor margins may be irregular or poorly appreciated on imaging, the ability to reference a model intraoperatively can minimize the complication rate and improve a higher rate of negative margins [8]. The use of patient-specific 3D printed models has also shown to lead to greater surgical accuracy, reduced operating room (OR) time, and better patient outcomes [9-11].

Cervical foraminotomy is a procedure used for the treatment of patients with unilateral radiculopathy. The procedure can be performed using a minimally invasive technique resulting in reduced blood loss, shorter hospital stays, and decreased post-operative medication [12]. For a posterior foraminotomy, intraoperative image guidance can be used to locate the correct surgical level, determine the foraminotomy size, and check the accuracy of decompression [13,14]. These benefits, however, can also lead to longer operating times [15]. Additionally, complete visualization of relevant anatomy remains difficult to obtain with the small incisions used. Therefore, having a 3D printed model of the spinal anatomy of interest may be helpful in planning and confirming the incision level and identifying potentially challenging anatomy not obvious by imaging alone. While the use of 3D models has been evaluated in multiple subspecialties within neurosurgery such as cerebrovascular and neuro-oncology, their application in cervical foraminotomy remains underexplored, despite the intricate nature of the procedure and possible complications due to proximity to critical neurovascular structures [16,17]. In a review of 3D printing in spine surgery, 3D models were used for pre-operative visualization for tumor resections, pedicle screw guides, osteotomy guides, and other implants [16]. To the best of our knowledge, the use of 3D models for cervical foraminotomy has not been previously published. Our study aims to assess its utility by comparing operative time, estimated blood loss, fluoroscopy time, and functional outcomes between patients undergoing cervical foraminotomies with 3D models and those without.

## Materials and methods

Following Institutional Review Board (IRB) approval, we conducted a retrospective case-controlled study. This study included patients who underwent cervical foraminotomies performed by a single surgeon at the Puget Sound Veterans Affairs Healthcare System between July 1, 2019, and December 31, 2021, where the surgeon requested a pre-operative patient-specific 3D model. A minimally invasive technique was used for all these patients.

Patients who underwent 3D model-assisted surgery were matched based on age and levels of foraminotomy with patients who underwent conventional cervical foraminotomies without pre-operative 3D models by the same surgeon during the same period. Controls were sequentially selected based on the date of surgery and levels of foraminotomy. Specifically, patients in the 3D model group who underwent single-level foraminotomies were matched to control patients who also underwent single-level foraminotomies. Patients who underwent two-level and three-level foraminotomies were matched accordingly. Patients with potential confounding comorbidities that may cause upper extremity weakness or paresthesia such as cervical spinal stenosis and a history of stroke were excluded from both groups. Exact matching of other comorbidities could not be achieved due to the small sample size and retrospective study design.

Patient records from the Computerized Patient Record System (CPRS) at the Puget Sound Veterans Affairs Healthcare System were retrospectively reviewed to collect data on patient demographics, including age and sex, body mass index (BMI), operative time, fluoroscopy time, intraoperative estimated blood loss volume, and functional outcomes. For functional outcomes, partial improvement was defined as improvement in sensory or motor functions, but not both. Improvement was defined as improvement in both sensory and motor. Resolved was defined as the complete resolution of symptoms.

Patient-specific 3D model creation

**Figure 1:**
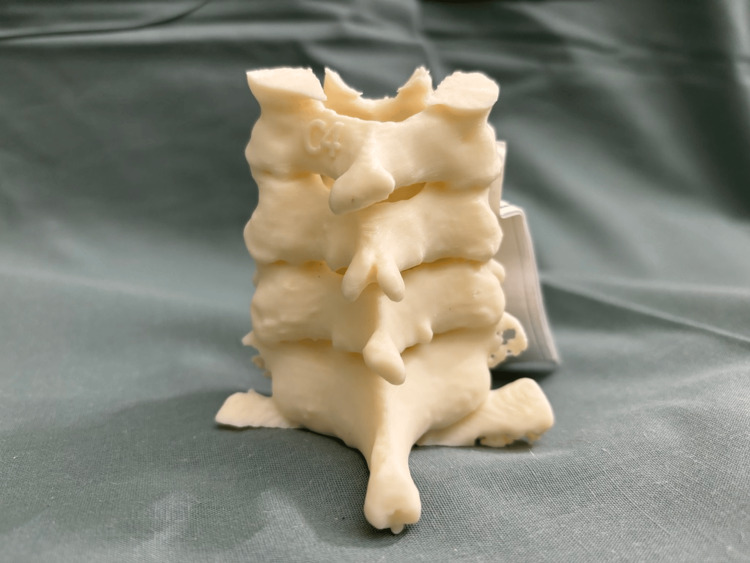
3D model of the cervical spine from C4 to C7 levels (posterior view) 3D: three-dimensional

**Figure 2:**
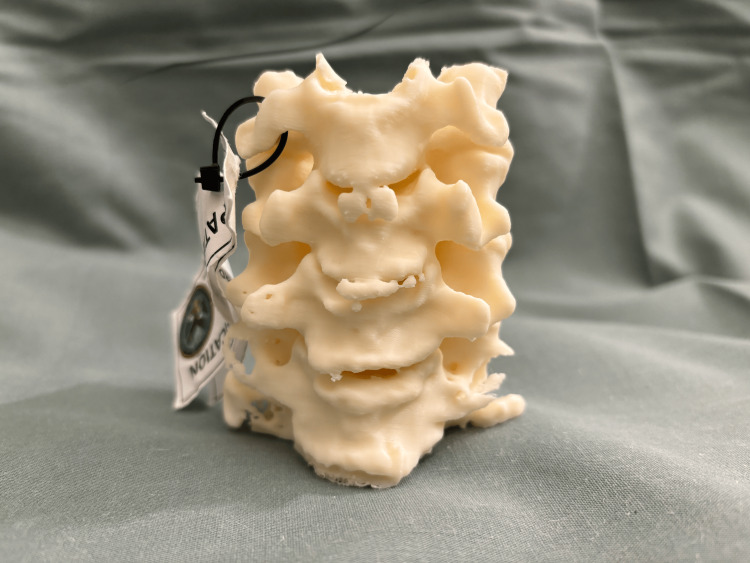
3D model of the cervical spine from C4 to C7 levels (anterior view) 3D: three-dimensional

**Figure 3:**
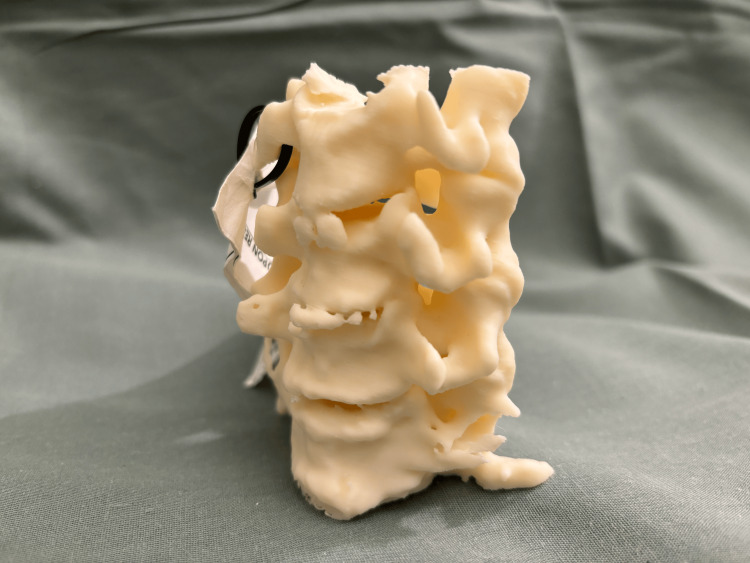
3D model of the cervical spine from C4 to C7 levels (anterolateral oblique view) 3D: three-dimensional

Patients’ CT images were obtained and processed into Digital Imaging and Communications in Medicine (DICOM) format. The cervical vertebrae were segmented into 3D models using MIMICS version 23.0 (Materialise, Interactive Medical Image Control System, Leuven, Belgium). These 3D anatomic models were then exported in Surface Tessellation Language (STL) format for 3D printing. Each model was printed at a 100% scale. The models then underwent postprocessing and completed a standard quality control process involving visual inspection, measurement against imaging data, and labeling to ensure consistency across cases. The quality control process was previously described by Bastawrous et al. [18]. An example 3D model is presented in Figures 1-3.

Cervical foraminotomy surgery

All surgeries in this study were performed by the same surgeon using the standard of care technique with or without pre-operative 3D models.

Statistical analyses

All statistical analyses were conducted using R statistical software (R version 4.2.3) [19]. Patient demographics were summarized using descriptive statistics, and the conventional and 3D model-assisted groups were compared using a two-sample t-test. Similarly, total operative time, estimated blood loss volume, and intraoperative fluoroscopy time were also compared using a two-sample t-test. Pearson's chi-squared test was used to compare postoperative functional outcomes. Data were reported as mean ± standard deviation. p<0.05 was considered statistically significant.

## Results

During the study period, patient-specific 3D cervical spine models were printed for nine patients undergoing foraminotomies. One of these patients was excluded from the final analysis due to concurrent scapular mass resection and cervical foraminotomy. Eight patients were selected as controls. There was no significant difference in age or BMI between patients undergoing standard of care operation compared to 3D model-assisted foraminotomy. The patient characteristics are summarized in Table 1.

**Table 1:** Summary of patient characteristics BMI: body mass index; SD: standard deviation; 3D: three-dimensional

	Patients undergoing standard of care operation (n=8)	Patients undergoing 3D model-assisted foraminotomies (n=8)	p-value
Mean age ± SD (y)	45 ± 8	53 ± 13	0.18
Male sex	8	8	1
BMI ± SD	30.9 ± 5.1	32.3 ± 4.5	0.57

.Total fluoroscopy time for 3D model-assisted surgeries was significantly lower than the controls (p<0.05). Total fluoroscopy time was 21.8 ± 8.7 minutes for 3D model-assisted surgeries and 49.0 ± 31.6 minutes for the control group.

While the average operative time for 3D model-assisted surgeries (236.4 ± 67.8 minutes) was greater than conventional surgeries (204.9 ± 26.4 minutes), there was no significant statistical difference (p=0.25). There was also no significant difference in estimated blood loss volume between 3D model-assisted surgeries (33.1 ± 29.8 mL) and conventional surgeries (21.3 ± 9.5 mL), p=0.31.

**Figure 4:**
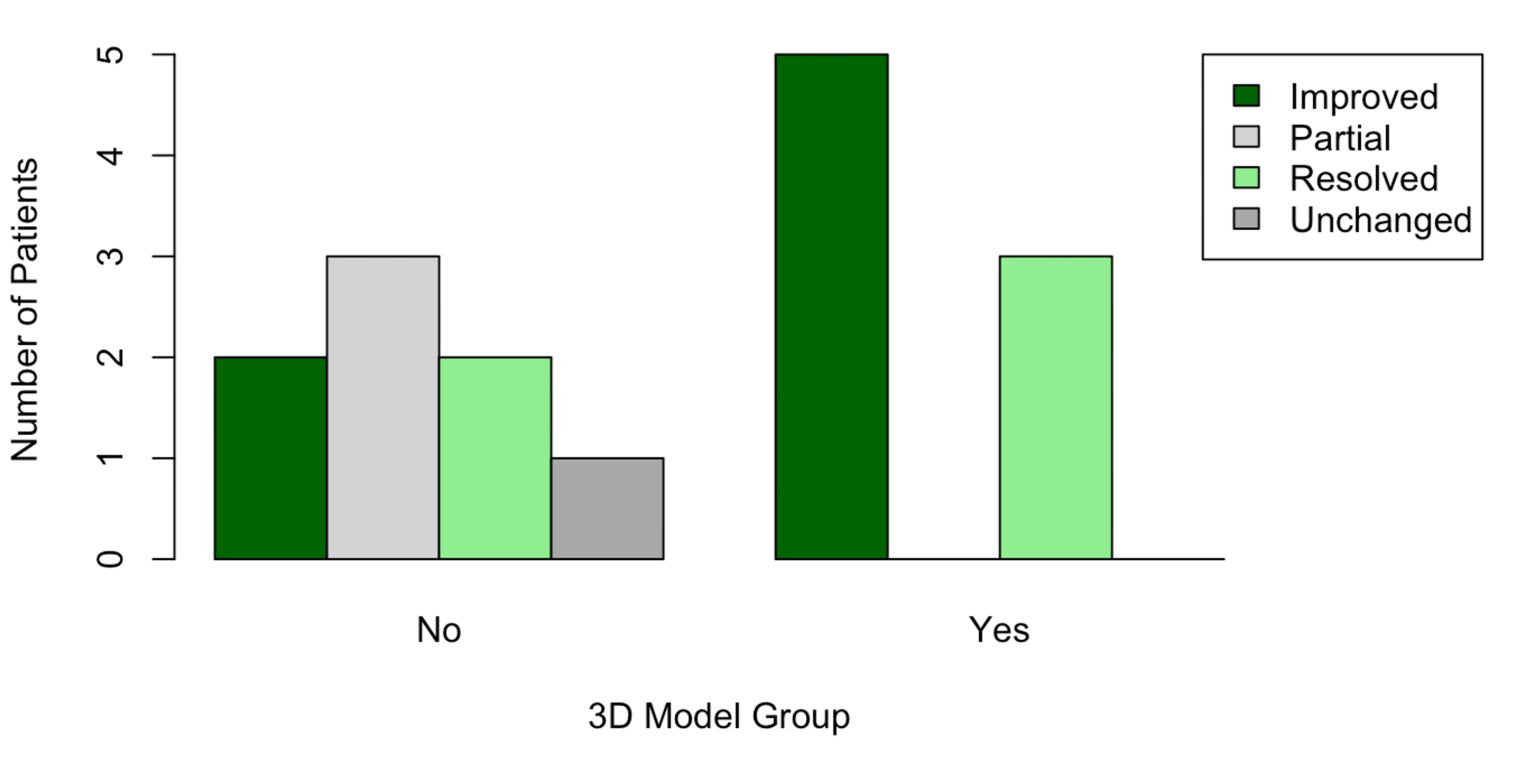
Functional outcome of patients whose foraminotomy was performed with 3D models compared to those without 3D: three-dimensional

There was no significant difference in functional outcome between 3D model-assisted and conventional surgeries (p=0.14), which could be due to the small sample size or patient heterogeneity. A greater proportion of patients undergoing surgery with 3D models, however, reported improved or resolved sensory and/or motor symptoms (Figure 4).

## Discussion

Cervical radiculopathy is a common condition with a reported prevalence ranging from 1.21 to 5.8 per 1,000 person-years [20]. An epidemiologic survey of patients with cervical radiculopathy found that at five years follow-up, 31.7% experienced symptom recurrence and 26% underwent surgical intervention for intractable pain, sensory deficit, or objective weakness [21]. No clear consensus guidelines exist regarding indications for surgical intervention or surgical approach to use for the treatment of cervical radiculopathy.

This study compares surgical and functional outcomes between patients undergoing 3D model-assisted and standard cervical foraminotomy without 3D models. While the use of 3D models for cervical spine surgeries has been reported for tumor resection planning and pedicle screw guides, this is the first study to investigate the use for cervical foraminotomy.

Our study demonstrated that cervical foraminotomies performed using 3D models significantly reduced fluoroscopy time compared to the conventional method which may reduce radiation-related risks including skin injury, fertility dysfunction, and radiation-induced malignancies. The surgeon reported that the use of 3D models increased his familiarity with the patient’s anatomy. This finding aligns with previously published research, which highlights that 3D models enhance the visualization of anatomical landmarks not easily appreciable on imaging studies and reduce radiation exposure [8,22-24]. The increased familiarity with the patient’s anatomy likely reduced the need for repetitive imaging to confirm anatomical details, thereby decreasing patients’ radiation exposure.

While fluoroscopy time was significantly lower with the use of 3D models, the average operative time was higher compared to the conventional approach, although this difference was not statistically significant. This increase in operative time was likely due to the 3D models being requested for more complex cases and/or the additional intraoperative steps taken to utilize the models effectively. Additionally, the surgeon also noted that his speed in performing foraminotomies progressively improved over time, partly due to the educational value of the 3D models, which serve as more than just an adjunct surgical tool [25-27].

Functional outcomes improved for nearly all patients undergoing either 3D model-assisted or standard approach operations. All patients who underwent 3D model-assisted operations experienced improvement or resolution of symptoms. However, approximately half of the patients in the control group reported only partial or unchanged symptoms. 

This study has several limitations, including its non-randomized retrospective design, small sample size, and reliance on a single surgeon’s experience. Only male patients were included in the study population, which is reflective of the Veterans Affairs’ patient population, but limits its generalizability. During the study duration, 3D models were typically requested for more complex cases based on the surgeon’s preference, which may have skewed findings. Future studies should aim for randomization and include a larger sample size to represent the population better and provide more accurate results. Additionally, expanding the use of 3D models to different surgeons and in a variety of hospital systems could help make the results more generalizable across surgeons with varying experience levels and across a broader patient population. While this study focused on perioperative results, future investigation could also include the use of the same patient-specific 3D model for simulation training, which could also potentially reduce operative time.

## Conclusions

Patient-specific 3D models have the potential to serve as valuable tools in enhancing the understanding of a patient’s unique anatomical structure, thereby potentially improving the outcomes of cervical foraminotomy. Our study has demonstrated that the use of these models significantly reduces fluoroscopy time. By minimizing the duration of fluoroscopic imaging, the radiation dose is consequently reduced, thereby enhancing patient safety and decreasing the risk of radiation-induced complications. This is particularly pertinent in complex cervical foraminotomy cases, where precision and safety are paramount. 

These findings underscore the dual utility of patient-specific 3D models as both surgical and educational instruments. In a surgical context, they provide surgeons with a detailed, tangible representation of the patient's anatomy, allowing for more precise planning and execution of the procedure without the need for prolonged fluoroscopic imaging.
